# Movement behaviors and cardiometabolic risk in schoolchildren

**DOI:** 10.1371/journal.pone.0207300

**Published:** 2018-11-14

**Authors:** Lidia Lucas-de la Cruz, Vicente Martínez-Vizcaíno, Jorge Cañete García-Prieto, Natalia Arias-Palencia, Ana Diez-Fernandez, Marta Milla-Tobarra, Blanca Notario-Pacheco

**Affiliations:** 1 Universidad de Castilla-La Mancha, Faculty of Nursing, Cuenca, Spain; 2 Universidad de Castilla-La Mancha, Social and Health Research Center, Cuenca, Spain; 3 Universidad Autónoma de Chile, Facultad de Ciencias de la Salud, Talca, Chile; 4 Universidad de Castilla-La Mancha, Faculty of Nursing, Albacete, Spain; 5 Universidad de Castilla-La Mancha, Faculty of Education, Cuenca, Spain; 6 Hospital Nuestra Señora del Prado, Talavera de la Reina, Toledo, Universidad de Castilla-La Mancha, Cuenca; University of Maiduguri College of Medical Sciences, NIGERIA

## Abstract

Growing evidence has accumulated in recent years showing that movement behaviors have important implications for health in children, especially for cardiovascular health, whose risk factors could track from childhood to adulthood. However, these findings are mixed and inconsistent in children. The aim of this study was to examine the relationship between different movement behaviors (sedentary behavior, physical activity and sleep duration) and cardiometabolic risk in schoolchildren. The study shows cross-sectional results of baseline measurement from 146 Spanish schoolchildren, aged 8-to-11 years old, participating in the MOVI-2 study. Movement behaviors were determined using accelerometry combined with self-reported sleep time. Cardiometabolic risk was assessed using a validated metabolic syndrome index. Logistic regression analysis showed that higher levels of vigorous physical activity (OR = 0.110, p = 0.004) and sleeping more than 9 hours (OR = 0.269, p = 0.015) could be protective factors against metabolic syndrome risk in children. ANCOVA analysis showed associations between vigorous physical activity and waist circumference (p < 0.001), and sleep time with insulin resistance (p = 0.017) and lipid profile (p = 0.035). No association was observed between light and moderate physical activity, sedentary behavior and metabolic syndrome (index and components). No statistically significant differences were found for blood pressure and any of the movement behaviors. Our data suggest that both the amount of vigorous physical activity accumulated and sleep duration are independently associated with higher cardiometabolic risk in children.

## Introduction

The definition of metabolic syndrome (MetS) is still a debatable issue in childhood, however, quantifying cardiometabolic risk (CMR) in this age-group is clinically important because of the growing prevalence of obesity. [1] MetS has been defined as a cluster of cardiovascular risk factors, including central obesity, high arterial blood pressure, hypertriglyceridemia, low high-density lipoprotein-cholesterol (HDL-c) and insulin resistance. [2] It is considered a predictor of coronary heart disease and type 2 diabetes among adults. [3, 4]

MetS prevalence has increased substantially in the pediatric population, with a median in total populations of 3.3% (range 0%-19.2%). [5] In Spain, a prevalence of around 19% has been observed in obese children. [6]

Growing evidence has accumulated in recent years showing that movement behaviors such as sedentary behavior (SB), moderate-vigorous physical activity (MVPA) or sleep duration (SD) have important implications for health in children, [7–17] especially for cardiovascular health. In this vein, a negative association between physical activity (PA) levels and CMR factors has been reported. [18] Furthermore, studies using accelerometer-derived measures of PA have evidenced greater improvements associated with the practice of vigorous physical activity (VPA) than those with lower intensity PA. [19–22]

Another movement behavior providing a key role in the association of CMR in children is sleep duration. Although the quality of evidence has been rated low, most studies have reported that short sleep duration negatively influences cardiometabolic risk. [15] Since adiposity and most cardiometabolic risk factors track from childhood to adulthood, [3, 23] a better understanding of the relationship between movement behaviors and CMR may have long-term implications for adults’ cardiovascular health.

As far as we know, there is no other study in Spain that jointly analyzed the movement behavior patterns and the CMR considering PA at different intensities (moderate and vigorous), highlighting the possible importance of VPA and sleep duration over other movement behaviors.

Accordingly, the objective of this study was to determine the influence of movement behaviors (SB, PA and SD) on cardiometabolic risk in 8-to 11-year-old Spanish children.

## Materials and methods

This is a cross-sectional study of baseline data from a cluster-randomized trial aiming to test the effectiveness of a PA program for the prevention of excess weight in schoolchildren (MOVI-2). [24] The study included 1158 schoolchildren, aged 8-to-11 years, from 20 public primary schools in the province of Cuenca, Spain. To participate, children had to be literate in Castilian Spanish, could not have any physical or mental disorder identified by parents or teachers which would prevent them doing PA, and could not suffer from any chronic disease that their paediatrician or family doctor considered would prevent them from participating in MOVI-2. The study was conducted between 2010–2011. Of the baseline sample, 210 participants (55.2%, girls) were randomly selected to wear an accelerometer. For this report, we used data from a sub-sample of 146 children (54.8% girls), from which we obtained complete data for the variables included in the study. This subsample size was calculated considering a difference between control group and intervention group of a 10% in the proportion of children meeting physical activity recommendations (0.05 significance level and 0.8 of statistical power). These children presented no differences in age, sex or parental socioeconomic status from the total sample of children participating in the trial.

The Clinical Research Ethics Committee of the Virgen de la Luz Hospital in Cuenca approved the study protocol (number PII1I09-0259-9898). The study follows the principles of the Declaration of Helsinki. [25] After obtaining approval from the principal and school council of each school, a letter was sent to all parents of children in fourth and fifth grade inviting them to a meeting at which the study objectives were outlined and written approval for their children’s participation was obtained. Informative talks, in which the schoolchildren were asked to collaborate, were then held class-by-class.

### Measurements

#### Anthropometric measurements

Waist circumference (WC) was determined by the average of two measurements taken with flexible tape at the waist (at the midpoint between the last rib and the iliac rest). Each variable was measured twice, and a third measurement was made when the difference between the first two was outside the range allowed for each measure. The average of the two closest measurements was utilized in the analyses.

#### Blood pressure

Diastolic and systolic blood pressure (DBP and SBP) were determined by the average of two measurements taken at an interval of 5 min, with the subject resting for at least 5 min before the first measurement. The participant was seated with their right arm placed in a semi-flexed position at heart level in a quiet, calm environment. Blood pressure was measured by an automated digital sphygmomanometer, the OMROM M5-I monitor (Omrom Healthcare Europe BV, Hoofddorp, Netherlands). Subsequently, the mean arterial pressure (MAP) was calculated using the following formula: DBP + [0.333 x (SBP–DBP)]. Trained nurses measured the anthropometric variables and blood pressure.

#### Biochemical assessments

Blood samples were taken under standardized conditions between 8:15 and 9:00 a.m., after at least 12 hours fasting by puncturing the cubital vein. Before the extraction, fasting was confirmed by the child and their parents. When it was anticipated that the transfer of samples to the laboratory would take longer than 75 minutes, they were centrifuged in situ and transferred refrigerated. Three aliquots of each sample were frozen, one for the determination of biochemical variables investigated in this study and the others for future analyses of which the parents were informed. [24]

The following biochemical parameters were determined: insulin, triglycerides (TG) and HDL-c. Lipid profiles were determined over a 48-hour period using a MODULAR DPP system from Roche Diagnostics, and insulin levels were assessed using an Immulite 2000 double system platform of Siemens. The coefficients of variation between batches were as follows: insulin, 2.47 to 3.34%; triglyceride, 1.12%; and HDL-c, 2.93 to 2.07%.

#### Cardiometabolic risk assessment

We calculated a cardiometabolic risk index (CMRI) as the sum of the age-sex standardized scores of WC, TG-to-HDL-c ratio, MAP, and fasting insulin. The validity of this index has been previously tested using confirmatory factor analysis. [26]

#### Physical activity

Physical activity was measured using the GMTI accelerometer model 7164 (ActiGraph, Shalimar, FL, USA). The children were instructed to position the monitor on their right hip for seven consecutive days. They were also instructed to wear the accelerometer at all times, including during the night, and only to remove it during water-based activities, i.e. swimming or having a shower or bath. The accelerometer was set to record PA data every minute (60s epoch). The KineSoft (v.3.3.2.0 software) was used to analyze the data. Sequences of 10 or more consecutive “zero” counts were considered to be non-wearing time and were excluded from the analyses. A minimum of four days’ recording (including at least one weekend day) with 10h of recorded time per day was set as the inclusion criteria. Time spent (min/day) at different intensities was calculated using the cut-off points proposed by Evenson et al.: [27] sedentary time (0–100 cpm), light PA (101–2295 cpm), moderate PA (2296–4011 cpm) and vigorous PA (≥4012 cpm). MVPA was calculated as the sum of time spent in moderate and vigorous PA.

#### Sleep duration

Sleep Duration (SD) was objectively assessed using the same accelerometer device used to determine physical activity. Actigraphy data were collected in 60-second epochs and with a 30-hertz sampling rate.

Furthermore, the parents and children were instructed to keep logs for bedtime (‘‘lights off” and trying to sleep) and waking time (‘‘lights on”) during the week the monitor was worn. These self-reported bedtimes and waking times were used to estimate accelerometer determined sleep duration, allowing a visually inspected window of up to 30 minutes with accelerometer-registered data to avoid large inaccuracies in parent-reported times. There were no missing sleep log data for children included in this study. Caregivers were asked to record their children’s sleep logs each morning on awakening and to make sure the sleep logs were complete. Research staff checked children’s sleep logs when caregivers returned the monitors. Information from sleep logs was not used to substitute missing actigraphy data.

At the end of the observation period, data were analyzed using the ActiLife 6 software (the ActiGraph 2012, ActiLife version 6); for this, we used the ‘Sadeh’ sleep algorithm validated for children. [28] More than eighty-five percent of individuals had valid activity and sleep registrations for seven days and nights.

### Statistical analyses

Analyses were performed using IBM SPSS Statistics 24 (SPSS, Inc., Chicago, IL) and the level of significance was set at p < 0.05.

Descriptive characteristics were presented as means (standard deviation). All variables were checked for normality using both graphical and statistical (Kolmogorov-Smirnov) procedures, and appropriate transformations were applied when necessary. Sex-related differences were tested using Student-t for independent samples.

A logistic regression model was used to examine movement behavior predictors of high cardiometabolic risk of MetS. Due to the low prevalence of MetS observed in our sample, CMRI for this model was dichotomized according to the cardiometabolic risk amount, and participants were classified as low-medium (quartiles 1–3), and high (4^th^ quartile) cardiometabolic risk. Sedentary behavior, moderate and vigorous PA were categorized into tertiles as low (<33 percentile), medium (33–66 percentile) and high (>66 percentile). In order to gain applicability of categories, sleep time was categorized as <9 hours per day; between 9 and 10 hours per day; and >10 hours per day. The reference category was the one with highest expected risk, except for sedentary behavior. The model was adjusted for age and sex.

ANCOVA analyses were performed to evaluate the mean differences between z-scores of movement behavior and 33^rd^ and 66^th^ percentile of waist circumference, Tg/HDL-c ratio, insulin, blood pressure and MetS index. All analyses were controlled for age and sex.

## Results

Table 1 shows the descriptive data of the sample. Overall, our results showed that boys had significantly higher MAP than girls (p = 0.036), and also achieved more physical activity minutes than girls, mainly in the most intense category of physical activity. The mean daily MVPA was 48.82 (24.50) min, and 33.6% of the sample achieved the international daily recommendation of ≥60 min/day. [29] These findings were similar to other recent studies. [30] However, when we analyzed these data by sex, only 18.75% of girls and 51.52% of boys in our study complied with the recommendations.

**Table 1:** Characteristics of the sample by sex.

Variables	Total (n = 146)	Boys (n = 66)	Girls (n = 80)	*p value*
**Age (years)**	9.40 (0.74)	9.35 (0.79)	9.44(0.69)	0.470
**Weight (kg)**	36.38 (9.01)	37.19 (10.16)	35.71 (7.95)	0.326
**BMI (kg/m**^**2**^**)**	18.71 (3.81)	19.31 (4.43)	18.22 (3.17)	0.085
**WC (cm)**	67.78 (9.80)	68.88 (11.22)	66.87 (8.42)	0.219
**HDL-c (mg/dL)**	61.65 (12.67)	62.53 (13.09)	60.92 (12.35)	0.444
**TG (mg/dL)**	66.63 (37.08)	64.98 (37.36)	67.99 (37.03)	0.628
**Insuline (mg/dL)**	8.09 (4.14)	7.70 (3.99)	8.42 (4.25)	0.300
**MAP (mmHg)**	75.48 (6.82)	76.78 (6.39)	74.40 (7.01)	0.036*
**CMRI**	0.09 (1.76)	0.24 (1.84)	-0.04 (1.69)	0.340
**SB (min/day)**	422.91 (141.76)	434.40 (134.53)	413.44 (147.63)	0.376
**LPA (min/day)**	384.47 (78.34)	398.10 (70.08)	373.23 (83.30)	0.056
**MPA (min/day)**	38.88 (18.86)	48.01 (19.41)	31.35 (14.70)	<0.001**
**VPA (min/day)**	9.94 (7.71)	12.23 (9.39)	8.04 (5.34)	0.001*
**MVPA (min/day)**	48.82 (24.50)	60.24 (26.27)	39.39 (18.32)	<0.001**
**Sleep Time (min)**	531.83 (32.24)	521.72 (32.83)	540.16 (29.42)	<0.001**

Values represent mean (standard deviation). BMI, body mass index; CMRI, cardiometabolic risk index; HDL-c, high-density lipoprotein cholesterol; LPA, light physical activity; MAP, mean arterial pressure; MPA, moderate physical activity; MVPA, moderate-vigorous physical activity; SB, sedentary behavior; TG, triglycerides; VPA, vigorous physical activity; WC, waist circumference.

* p<0.05

** p<0.001.

Regarding the average nocturnal sleep time, 84.25% of the children (56.91% girls), complied with the recommendations of the National Sleep Foundation, which states that a child between 6 and 13 years of age should sleep between 9–11 hours per night to maximize overall health and well-being. [31]

Examining the relationship between movement behavior and the risk of MetS, after adjusting for age and sex, a decreasing dose-response risk was observed with increasing VPA (OR 0.204, 95% CI: 0.058–0.719 for 3.21–19.00 min/d; OR 0.110, 95% CI: 0.025–0.487 for >19.00 min/d) and sleep time (OR 0.313, 95% CI:0.104–0.946 for 9–10 h/d; OR 0.269, 95% CI: 0.093–0.776 for >10 h/d). However, no significant association was found with MPA, sedentary behavior and MetS risk. (Table 2)

**Table 2:** Independent relationship between movement behavior and cardiometabolic risk.

Movement Behaviors	Duration	n	OR (CI 95%)	p value
Sleep	< 9 h/d	48	**Ref.**	
	9–10 h/d	49	0.313 (0.104–0.946)	**0.039**
	> 10 h/d	49	0.269 (0.093–0.776)	**0.015**
SB	< 4.86 h/d	48	**Ref.**	
	4.86–9.42 h/d	49	2.486 (0.845–7.315)	0.098
	>9.42 h/d	49	1.955 (0.655–5.835)	0.230
MPA	< 19.72 min/d	49	**Ref.**	
	19.72–60.45 min/d	48	1.768 (0.517–6.043)	0.363
	> 60.45 min/d	49	3.197 (0.858–17.875)	0.078
VPA	< 3.21 min/d	48	**Ref.**	
	3.21–19.00 min/d	51	0.204 (0.058–0.719)	**0.013**
	> 19.00 min/d	47	0.110 (0.025–0.487)	**0.004**

CI, confidence interval; MPA, moderate physical activity; n, number; OR, odds ratio; Ref., reference; SB, sedentary behavior; VPA, vigorous physical activity.

**Fig 1:**
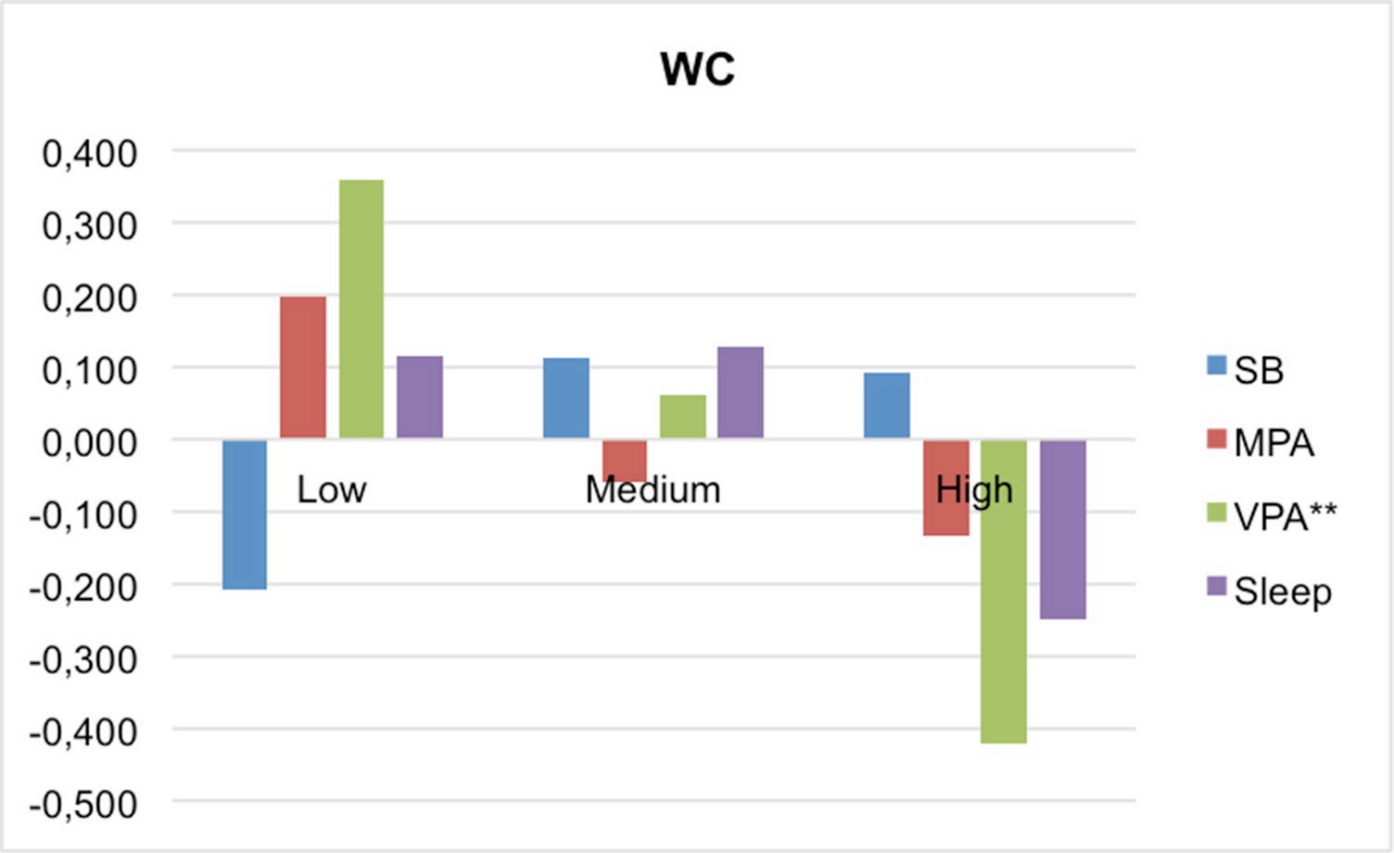
Mean differences in movement behaviors by WC categories. MPA, moderate physical activity; SB, sedentary behavior; VPA, vigorous physical activity; WC, waist circumference. **p<0.001.

**Fig 2:**
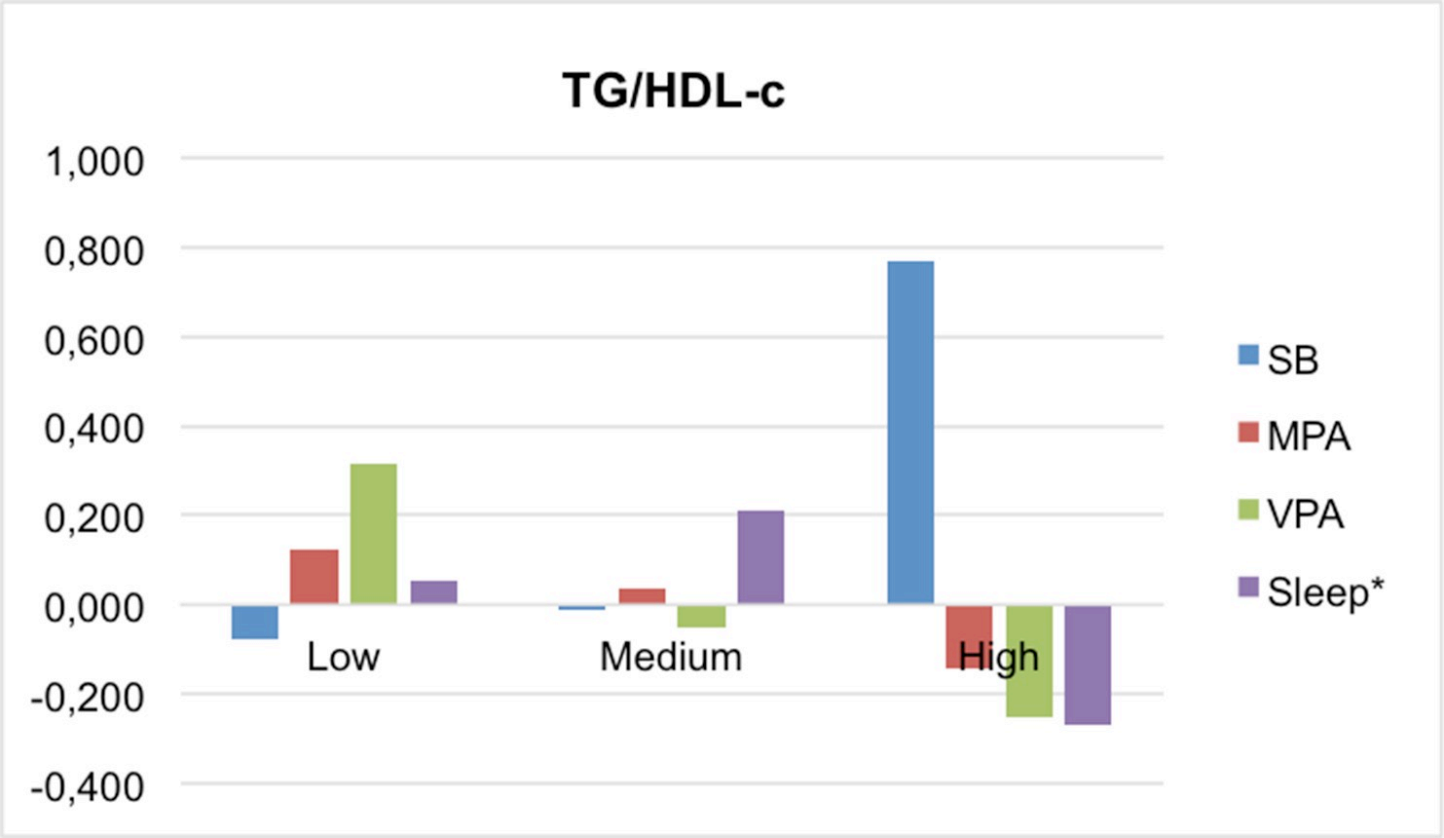
Mean differences in movement behaviors by TG/HDL-c categories. HDL-c, high density lipoprotein–cholesterol; MPA, moderate physical activity; SB, sedentary behavior; TG, triglycerides; VPA, vigorous physical activity. *p<0.05.

**Fig 3:**
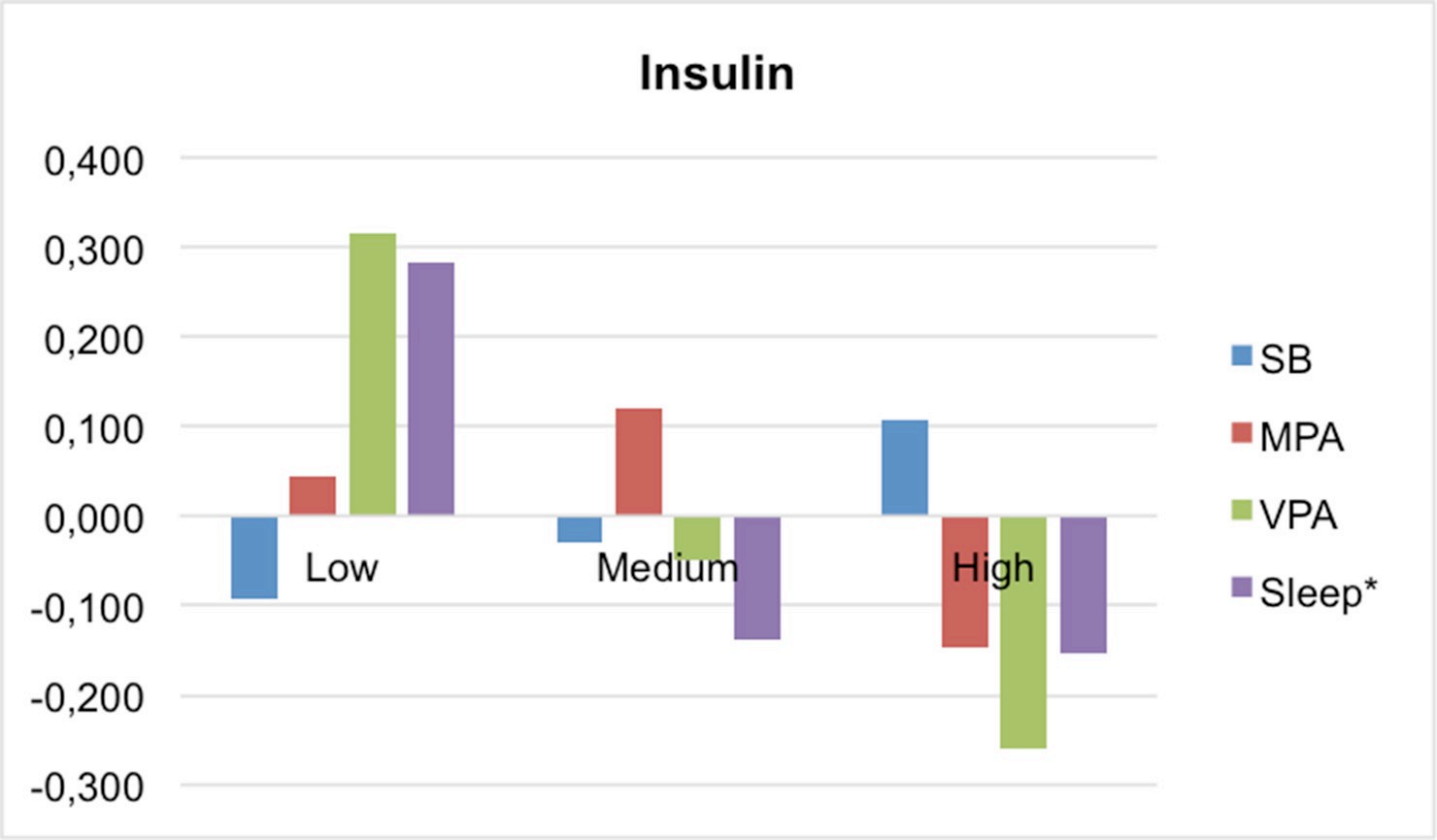
Mean differences in movement behaviors by fasting insulin categories. MPA, moderate physical activity; SB, sedentary behavior; VPA, vigorous physical activity. *p<0.05.

**Fig 4:**
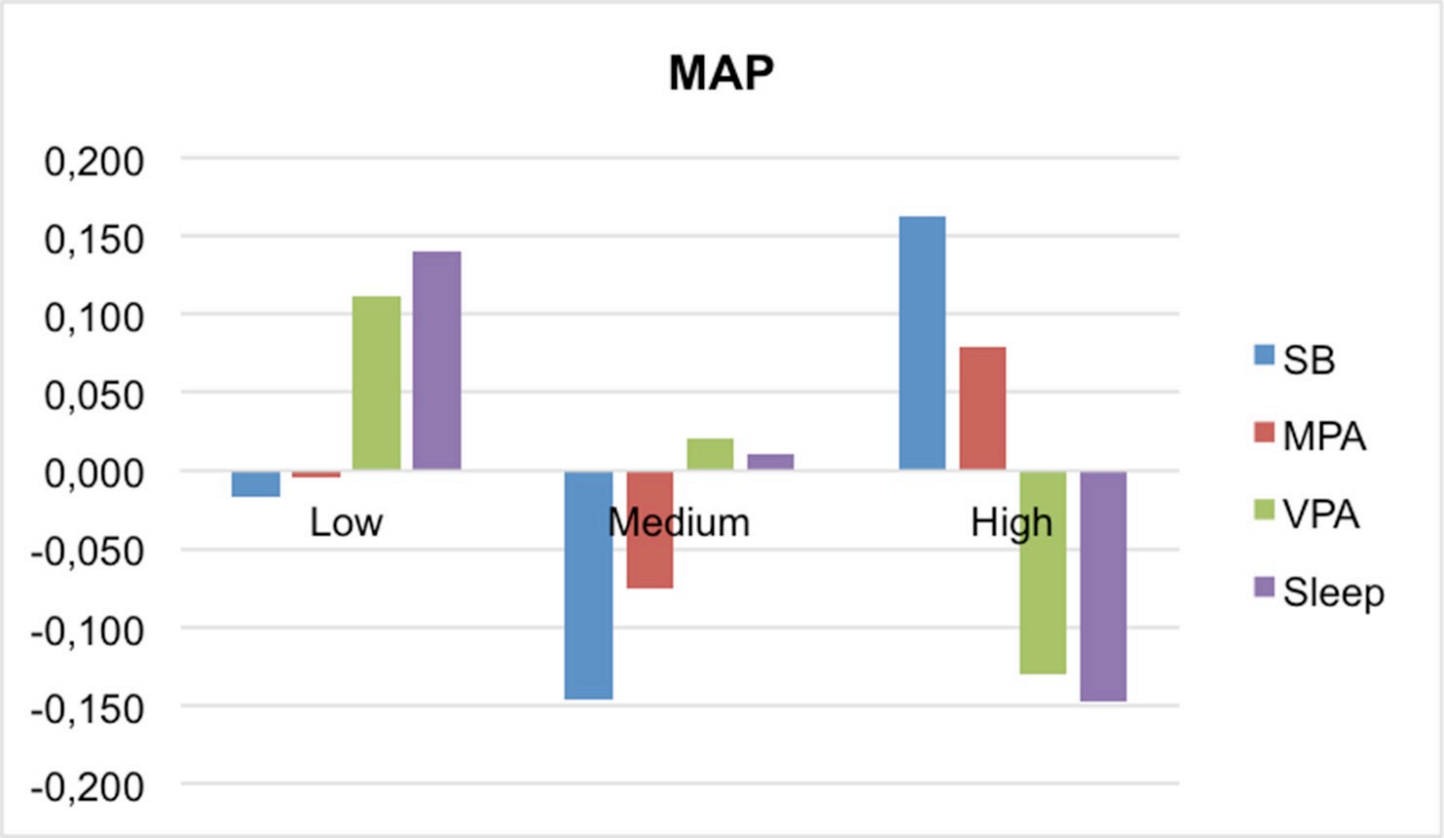
Mean differences in movement behaviors by MAP categories. MAP, mean arterial pressure; MPA, moderate physical activity; SB, sedentary behavior; VPA, vigorous physical activity.

Figs 1–4 present the mean differences between the z-scores of movement behaviors and percentile of each MetS component (waist-circumference, TG/HDL-c ratio, insulin and MAP, respectively). In general, children in the higher percentile of the cardiometabolic risk component presented less PA, slept fewer hours and spent more time on sedentary behavior. Statistically significant differences were found between waist circumference and VPA (p<0,001), and TG/HDL-c and insulin with sleep time (p = 0.035; p = 0.017, respectively). In the case of MAP, neither of the movement behaviors showed associations.

**Fig 5:**
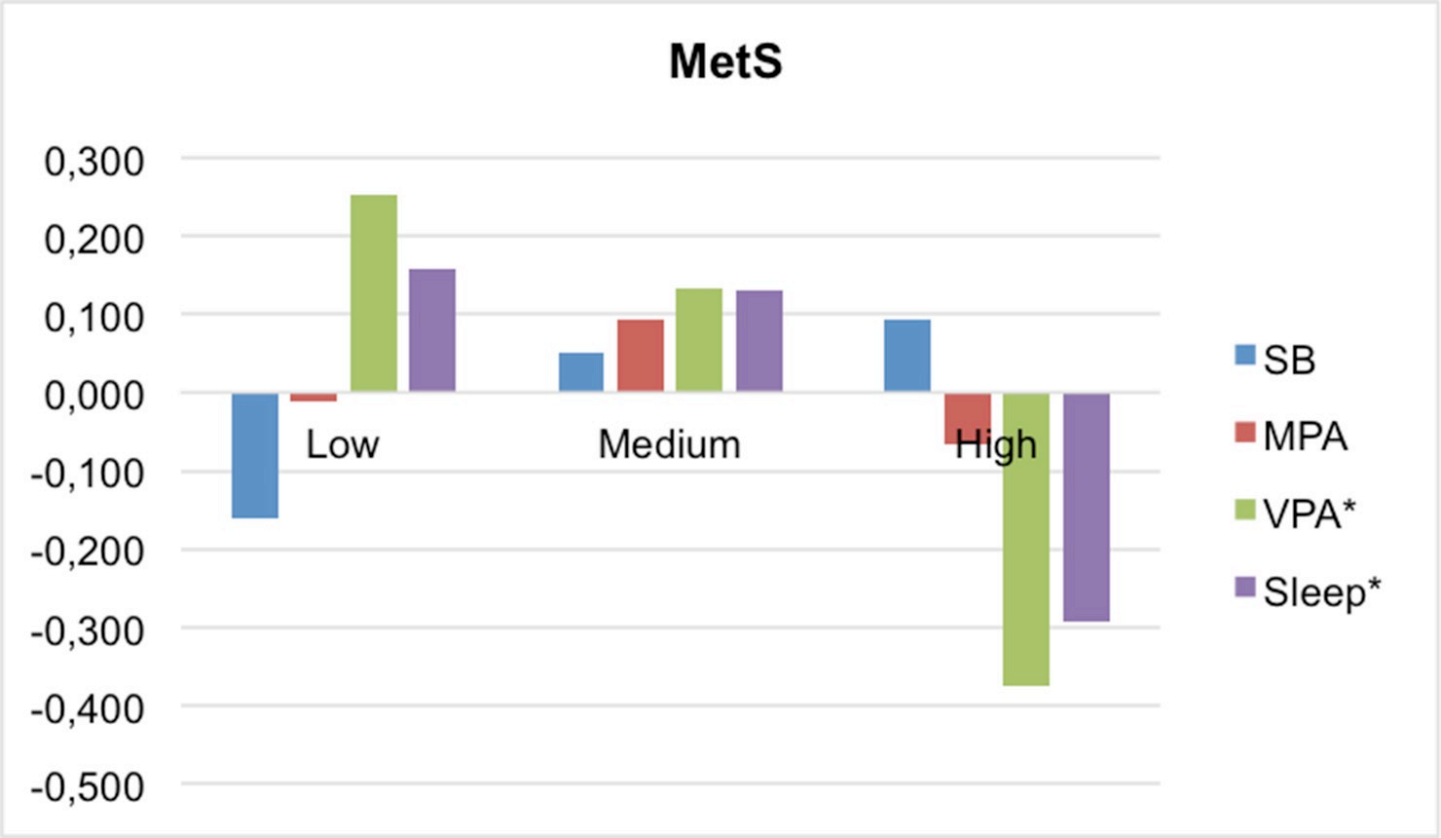
Mean differences in movement behaviors by MetS index categories. MetS, metabolic syndrome; MPA, moderate physical activity; SB, sedentary behavior; VPA, vigorous physical activity. *p<0.05.

Comparing movement behavior between MetS index categories, higher levels of CMRI are associated with lower time spent in VPA (p = 0.004) and sleep (p = 0.045). (Fig 5)

## Discussion

This study examines the association between movement behavior and MetS in children. The findings suggest that cardiometabolic risk decreases in a dose-response relationship with higher time spent on sleep and vigorous PA, but not with increasing moderate PA, in the line with previous research. [18–21, 32–36]

Our data show that approximately 20 min of vigorous PA are associated with a 94% reduction in cardiometabolic risk. These results evidence the key role of vigorous PA in the prevention of MetS risk in children and therefore, efforts should be made to increase the volume of vigorous PA children engaged, instead of light or moderate PA, as suggested in other studies. [11, 34]

When we separately analyzed the influence of physical activity on each of the components of cardiometabolic risk, we only observed significant reductions in VPA time for the children with higher WC. It should also be noted that vigorous PA is strongly associated with waist circumference, which is a valid measurement for estimating children’s and adolescents’ total and abdominal visceral fat [37] and is suggested as a noninvasive approach that could be used as a prescreening tool for classifying children with cardiometabolic risk. [38]

The positive association between sedentary behavior measured with accelerometers and cardiometabolic risk in children population is controversial.

The children in our study spent 46.6% of their waking hours in sedentary behavior. Moreover, a high percentage of children do not meet the internationally recommended levels of daily PA. Only 33.6% of children achieve at least 60 min of daily MVPA. Our results are similar to those of another recent study that reported a great percentage of children in similar ages who did not meet the recommended levels. [30]

Although the amount of sedentary time is notable, it was not associated with the risk of MetS or with the MetS components in our study. While some authors have not observed these differences, [11, 34] other cross-sectional studies show this positive relationship between the volume of sedentary time and MetS. [20, 39] The discrepancies between studies could be due to the differences in the cardiometabolic risk score assessment. Conversely, among adults, the volume of SB, as measured objectively by accelerometers, has been associated with clustering and individual CMRF, independently of other confounders. [40, 41] Arguably, these differences between children and adults might be explained by a physiological difference in the way SB impacts health in each one; and possibly, there are differences in the way of measuring these patterns in adults and youths.

Furthermore, our data suggest that sleeping more than 9 hours per day is a protective factor against risk of MetS, and the children in the higher tertile of insulin and TG/HDL-c are those who slept fewest hours. Despite considerably less data being available examining the association between insufficient sleep and insulin resistance in children, several studies have shown a positive association in obese children but not in normal-weight children. [42] As for the lipid profile, evidence suggests that sleep duration (short and/or long) may be associated with dyslipidemia in children, but that this association may be mediated by obesity. [42] Our results showed that the average of both HDL-c (m = 52.69) and TG (m = 106.45) was much higher in the obese children (n = 11) than the average of the total sample. In terms of waist circumference, several studies have reported that short sleep in childhood is associated with a higher waist circumference. [43, 44] Our results show no significant differences regarding sleep between waist circumference categories; however, it can be observed graphically that the children in the higher tertile are those with lower recorded sleep duration. In addition, our data are in line with the lack of consistency in the impact of sleep deficit on blood pressure in children, whereas in adolescents and adults it is clearer that chronic sleep deficit is associated with an increase in blood pressure. [42]

Furthermore, the average sleep time in our sample was 8h 58min, slightly lower than the average recorded in the last National Health Survey (2011–2012) showing data on sleep duration, which was 9.2 hours in children between 5 and 14 years. A high percentage of our sample (84.25%) meets the recommended levels proposed by the National Sleep Foundation regarding sleep duration (9-11h). However, the American Academy of Sleep Medicine recommends that a child between 5 and 12 years should sleep between 10 and 11 hours. If we take these recommendations into account, only 22.6% of our children (n = 33) slept 10 hours or more.

### Limitations and strengths

Our study has some limitations that should be acknowledged. First, its cross-sectional nature prevents us from making causality inferences. Although we have controlled for the different patterns of movement behavior in multivariate models, as well as for age and sex, some potential unmeasured confounders may be influencing our results; for example, we were not able to adjust for pubertal development, a factor influencing physiological processes. [45] The accelerometers used may have had limited sensitivity in differentiating between some types of movement; for example, in order to have a good estimation of sleep duration, a longer duration of epochs was used, and as a consequence the amount of vigorous activity could be have been underestimated. [46]

Third, due to the sample size, and because, as has been described in other studies, [33] the greater variability of time spent on vigorous-intensity physical activity in boys relative to girls, we did not analyze our results by gender, and some differences in the predictive ability of the different movement behaviors should not be ruled out.

However, despite these limitations, this study has significant strengths such as the use of an objective method to measure PA and sleep or the separate analysis of moderate and vigorous PA, which have allowed us to highlight the importance of VPA along with sleep duration.

## Conclusions

Our data have clinical and public health significance because, although efforts have traditionally been made to encourage children to accumulate a minimum of 60 min of MVPA, our findings suggest that only VPA has a significant influence on reducing cardiometabolic risk. Furthermore, our data suggest that children that sleep more than 10 hours reduce their cardiometabolic risk by 75%. However, more longitudinal research is needed to better understand the relationship between patterns of sleep and activity/movement behaviors and cardio-metabolic health at early ages.
